# Supplementary material for: Electronic signatures of Lorentzian dynamics and charge fluctuations in lithiated graphite structures
Source: Nat Commun. 2023 Apr 21;14:2291. doi: 10.1038/s41467-023-37857-3 (PMC10121681; doi:10.1038/s41467-023-37857-3)
Supplement: Supplementary file 1 — Supporting Information [file 41467_2023_37857_MOESM1_ESM.pdf]

Supplementary Information

Electronic signatures of Lorentzian dynamics and charge fluctuations in lithiated graphite structures

Sasawat Jamnuch and Tod A Pascal

Table of Contents

Supplementary Methods 2

Supplementary Tables 3

Supplementary Figures 5

Supplementary Methods

Supplementary Methods 1: Lorentzian fitting of XAS spectra

The two main XAS peaks at the π* and σ* resonances were fitted to Lorentzian functions, centered around a maxima:

$$L(x) = A\frac{w}{(x-x_0)^2+w^2}$$

where *A* is the amplitude, x_0 is the center and *w* is the width. The resulting Lorentzian fits were then used to find the area under the curve and the peak ratio, in Supplementary Table 1 below.

Supplementary Tables

Supplementary Table 1: Lorentzian fitting parameters.

	σ*		π*	
	**Amplitude[a]**	**Width (eV)**	**Amplitude**	**Width (eV)**
**Experiment**				
Stage I	0.69	0.67	3.26	3.16
Stage II	0.79	0.52	3.16	3.13
Stage III	0.87	0.49	3.03	2.94
Graphite	1.00	0.55	2.64	2.54
**Theory – Crystal Structure**				
Stage I	0.45	0.39	2.72	3.54
Stage II	0.60	0.43	1.44	2.23
Stage III	0.80	0.48	3.17	2.23
Graphite	1.00	0.40	3.42	1.51
**Theory – MD structures**				
Stage I	0.62	0.48	3.75	2.02
Stage II	0.82	0.52	3.27	1.23
Stage III	0.89	0.55	2.85	1.54
Graphite	1.00	0.46	2.97	2.11

[a]All amplitudes are scaled, assuming a value of 1 for Graphite

Supplementary Table 2: Distribution analysis of in-plane and out-of-plane Li atom displacement with staging

Li-GIC	Mean		Standard deviation		Skewness		Kurtosis	
	In-plane	Out of plane	In-plane	Out of plane	In-plane	Out of plane	In-plane	Out of plane
Stage I	0.000	0.000	0.119	0.049	-0.009	0.005	2.922	2.971
Stage II	-0.000	-0.000	0.182	0.090	-0.012	-0.135	2.786	5.216
Stage III	0.002	-0.001	0.132	0.080	0.069	0.009	3.241	3.632

Supplementary Table 3: Distribution analysis of C atom charge fluctuations with staging

Li-GIC	Mean	Standard deviation	Skewness	Kurtosis
Stage I	-0.144	0.036	0.119	2.755
Stage II	-0.072	0.040	0.218	2.801
Stage III	-0.029	0.083	-0.222	3.019
Graphite	-0.000	0.100	-0.081	2.680

Supplementary Table 4: Lattice parameters of graphite and LI-GICs supercell employed in the simulation.

Li-GIC	a (Å)	c (Å)	Volume of supercell (Å^3)
Stage I	12.948	10.772	1564.070
Stage II	12.840	13.485	1910.011
Stage III	12.820	9.770	1418.535
Graphite	7.3680	20.088	944.427

Supplementary Figures

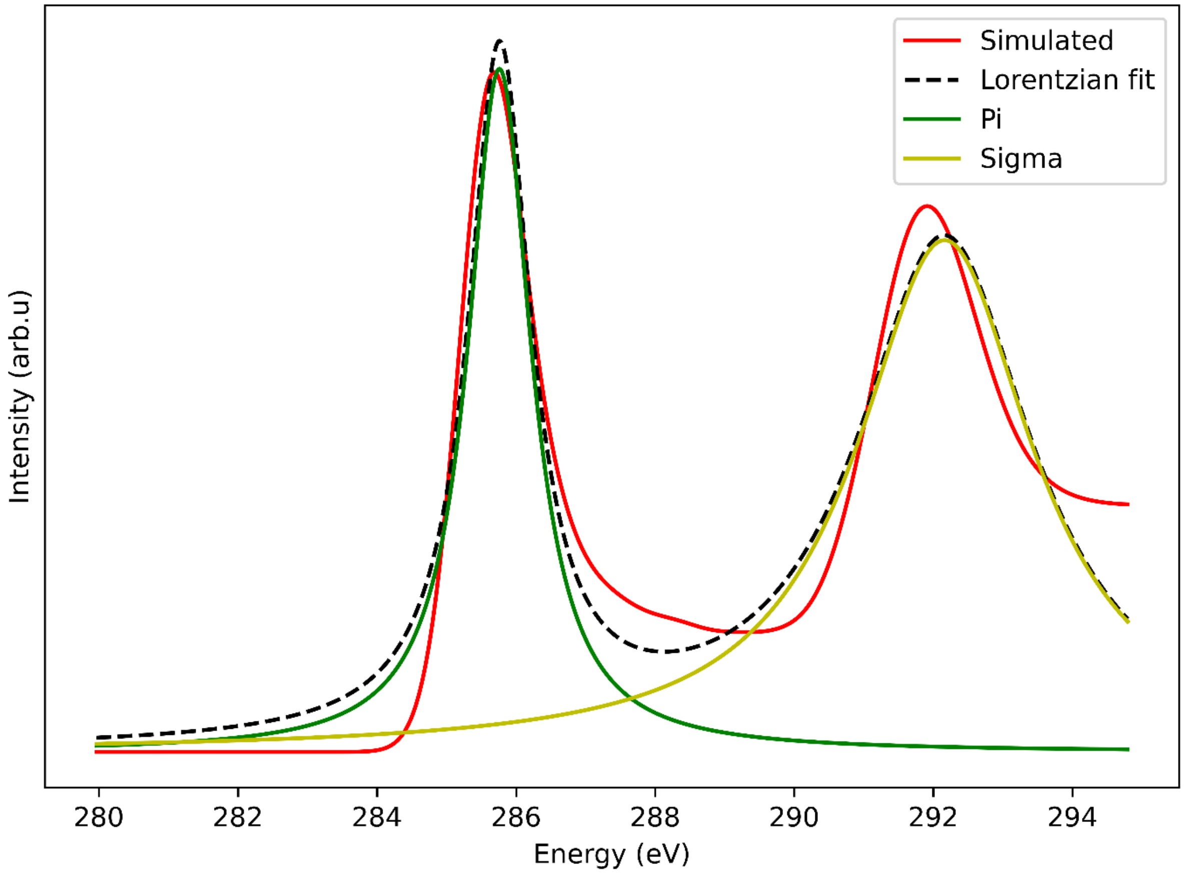

Supplementary Figure 1: Example of Lorentzian fitting to the LiC_{18} simulated spectra.

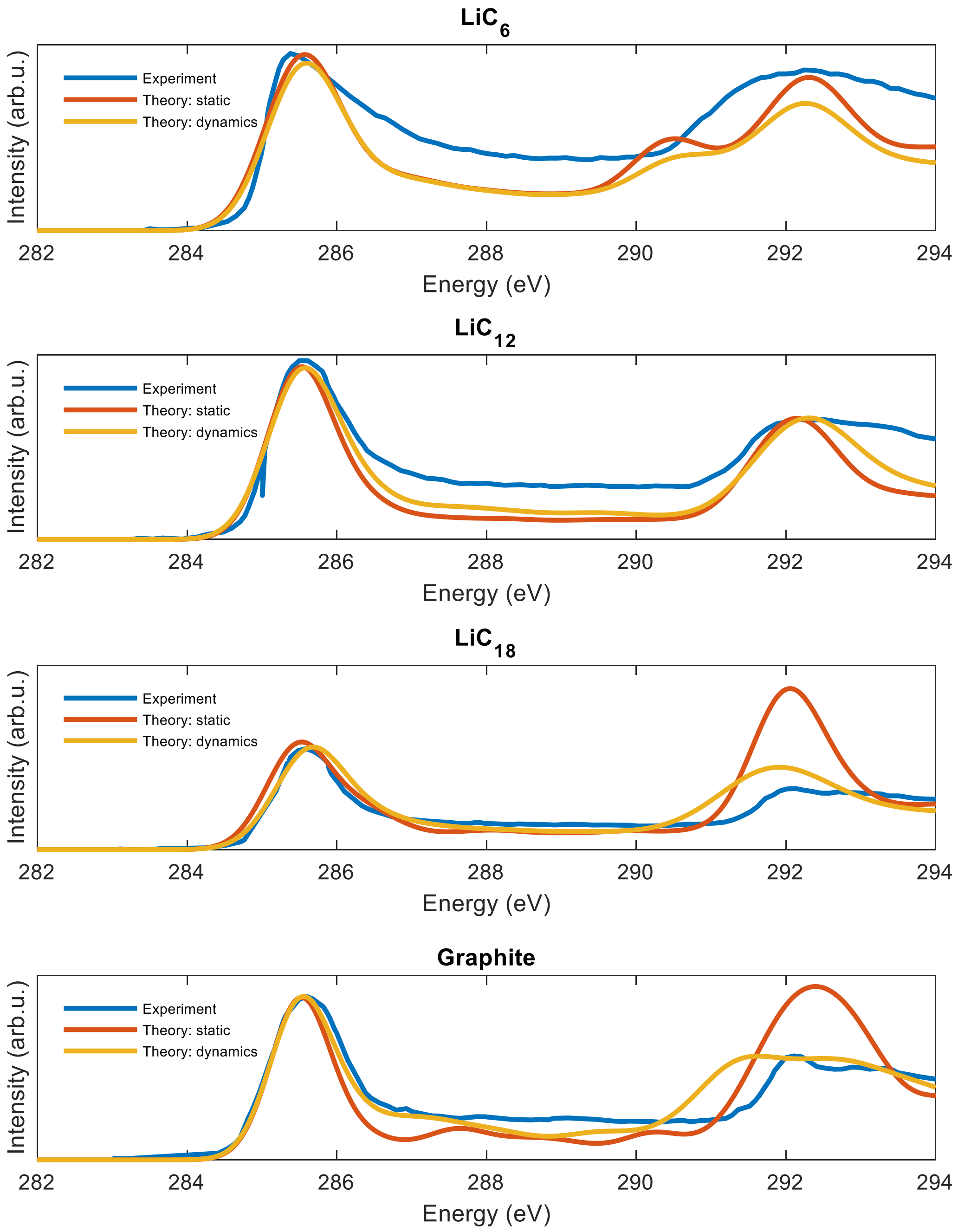

Supplementary Figure 2: Comparison between experimental and simulated XAS of graphite and Li-GICs at the Carbon K-edge, comparing the simulated spectrum based on the pristine crystal structure and MD structure. A dilation factor of 1.05 is used on the LiC_6 and LiC_{12} to match the second peak at sigma excitation.

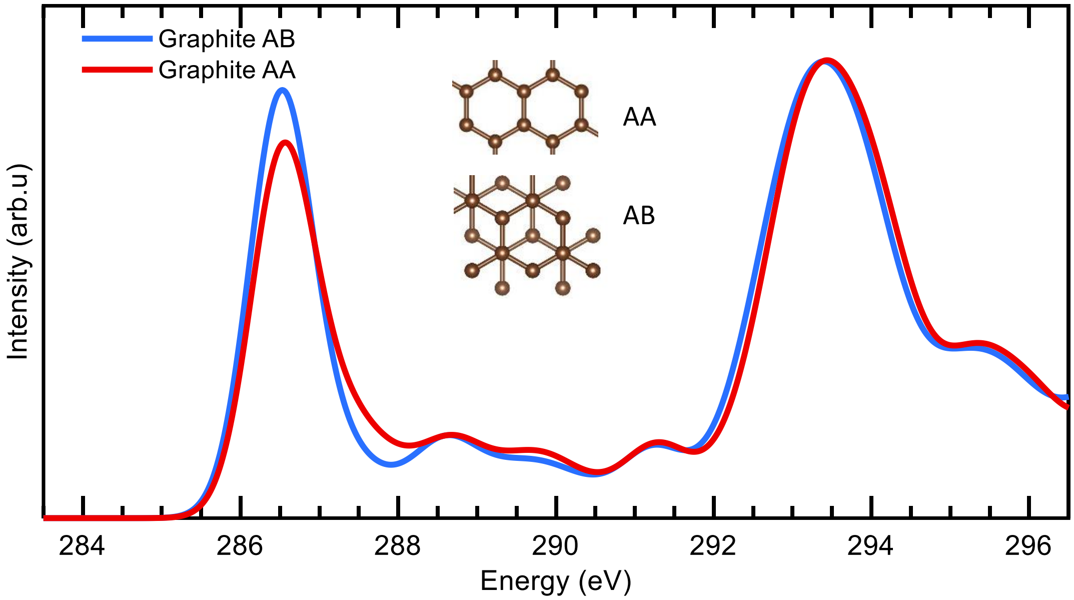

Supplementary Figure 3: Simulated XAS of graphite Carbon K-edge comparison between AA and AB stacked graphite.

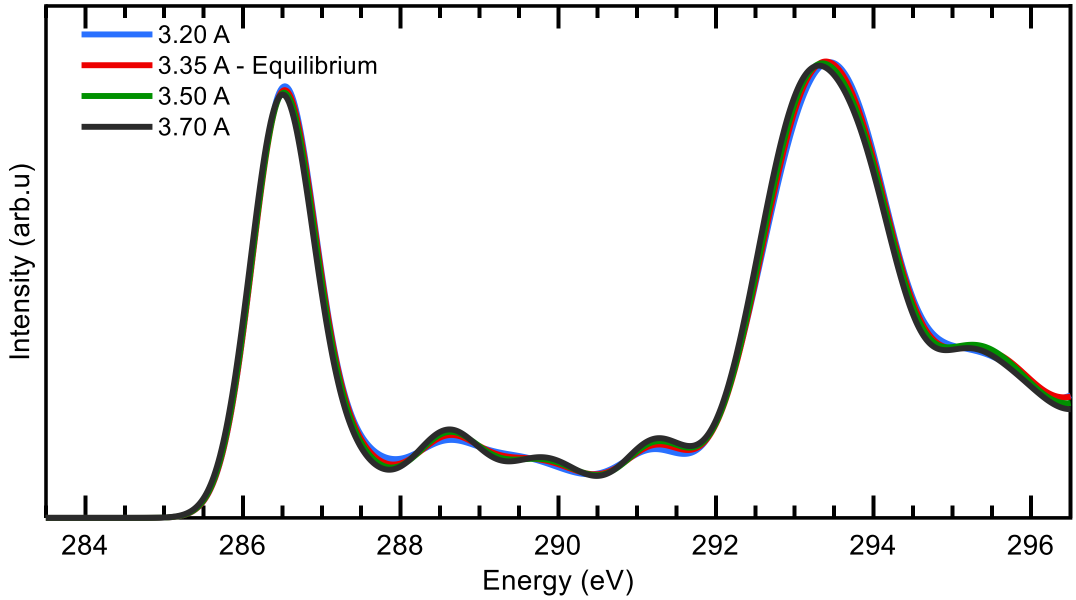

Supplementary Figure 4 Simulated XAS of graphite at the Carbon K-edge as a function of sheet-sheet interlayer distance.

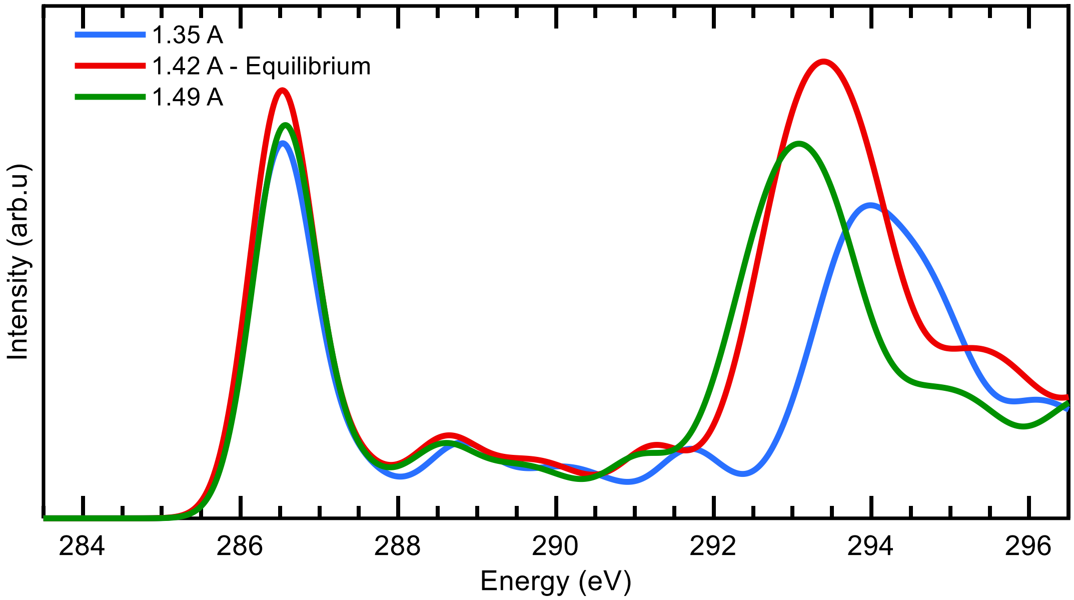

Supplementary Figure 5: XAS of AB stacked graphite as a function of in-place lattice constant

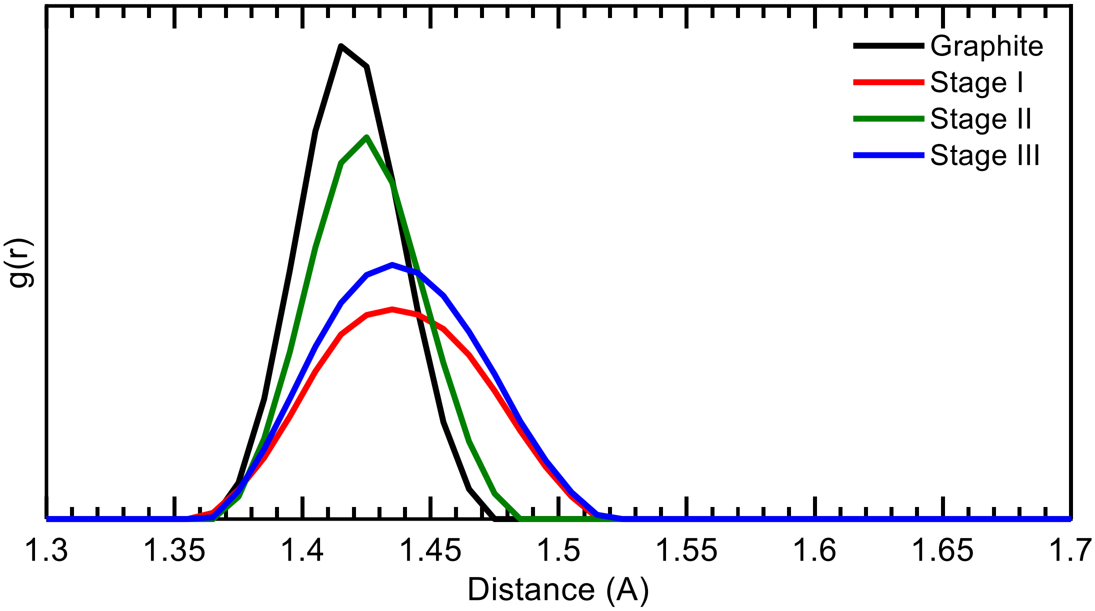

Supplementary Figure 6: Radial distribution function of graphite and Li-GICs

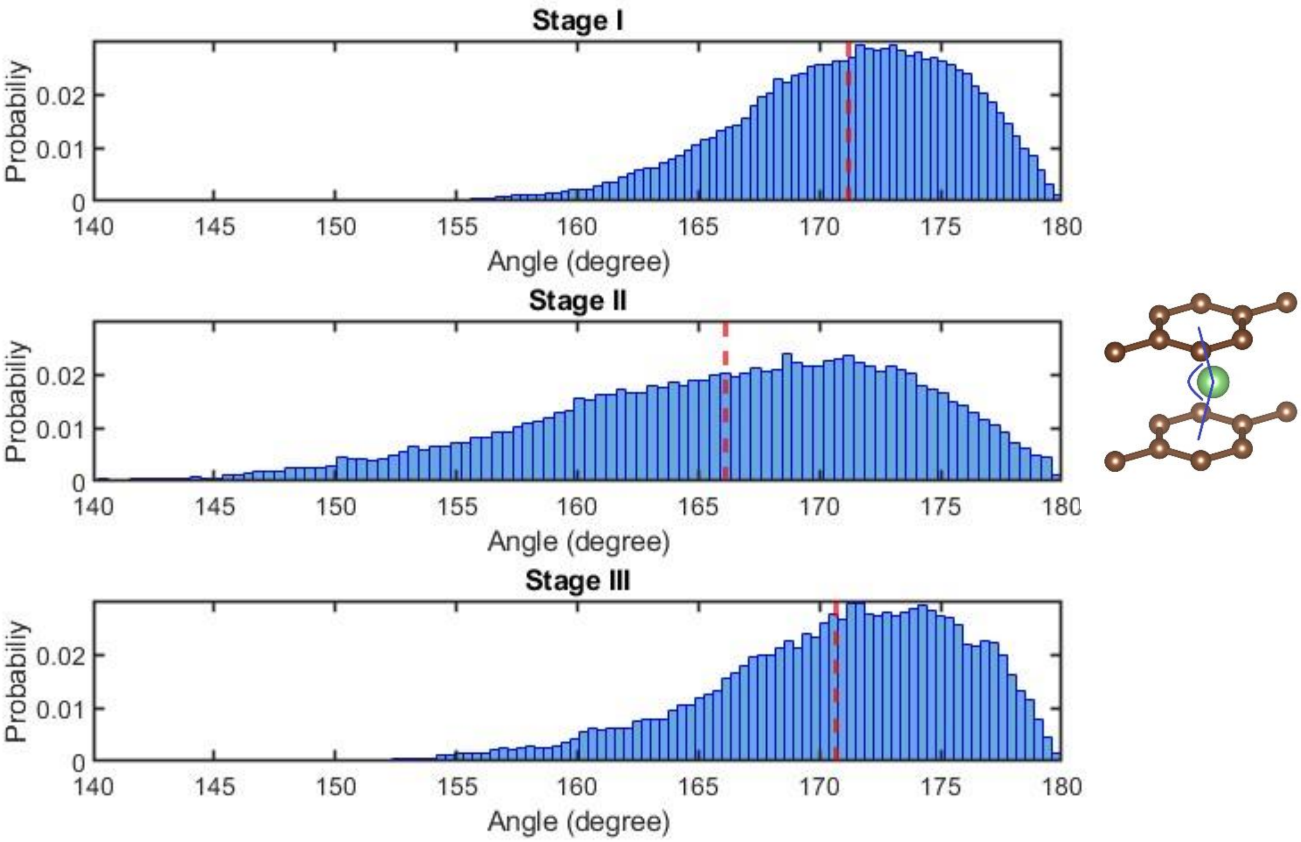

Supplementary Figure 7: Angle distribution of carbon hexagons and Li atom in Li-GICs

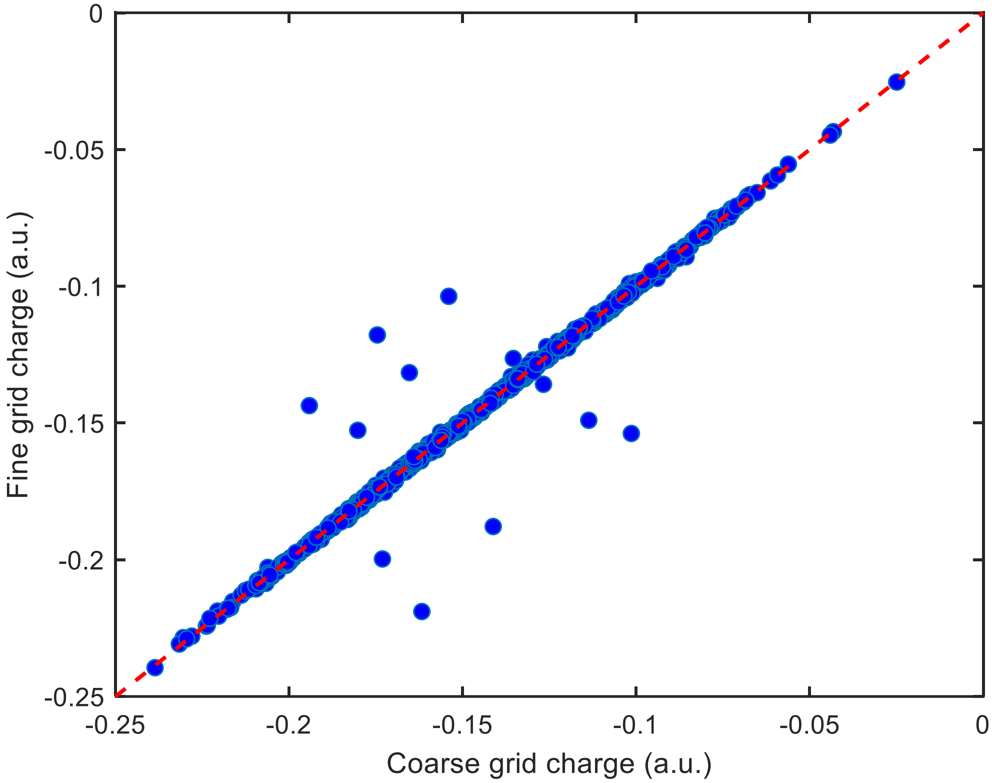

Supplementary Figure 8: Bader charge analysis comparison between coarse and fine k-point grid in LiC_6.

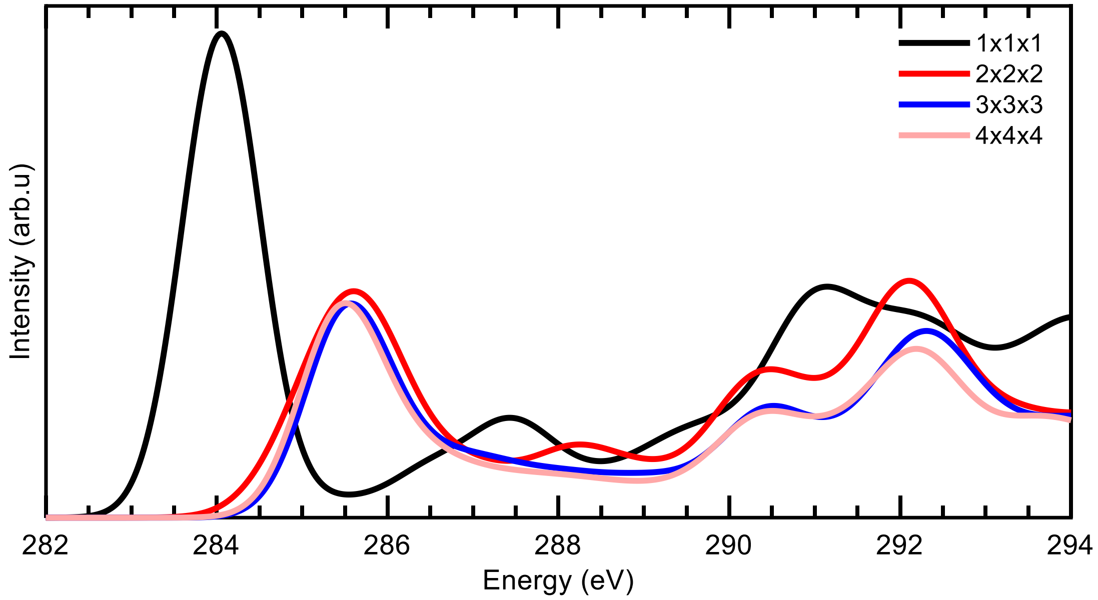

Supplementary Figure 9: XAS convergence as a function of supercell size in LiC_6. Only by expanding the supercell to 3x3x3 of the primitive cell that the cell lattices become larger than 10 Å that the self-interaction due to artificial periodicity is minimized. Note that at primitive cell, the peak position and intensity significantly deviated from expected behavior. The 3x3x3 and 4x4x4 have comparable spectra at the cost of approximate 16.5 times more computational cost.

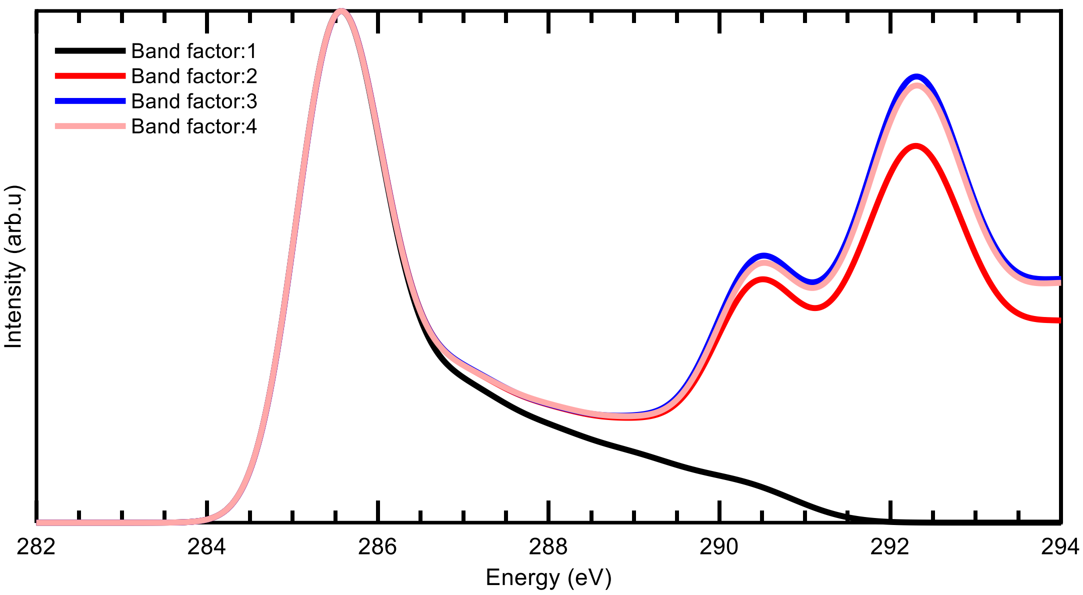

Supplementary Figure 10: XAS convergence as a function of the empty bands factor in LiC_6. Here, the factor represents the number of empty bands added to the NSCF calculation as a multiple of occupied bands.

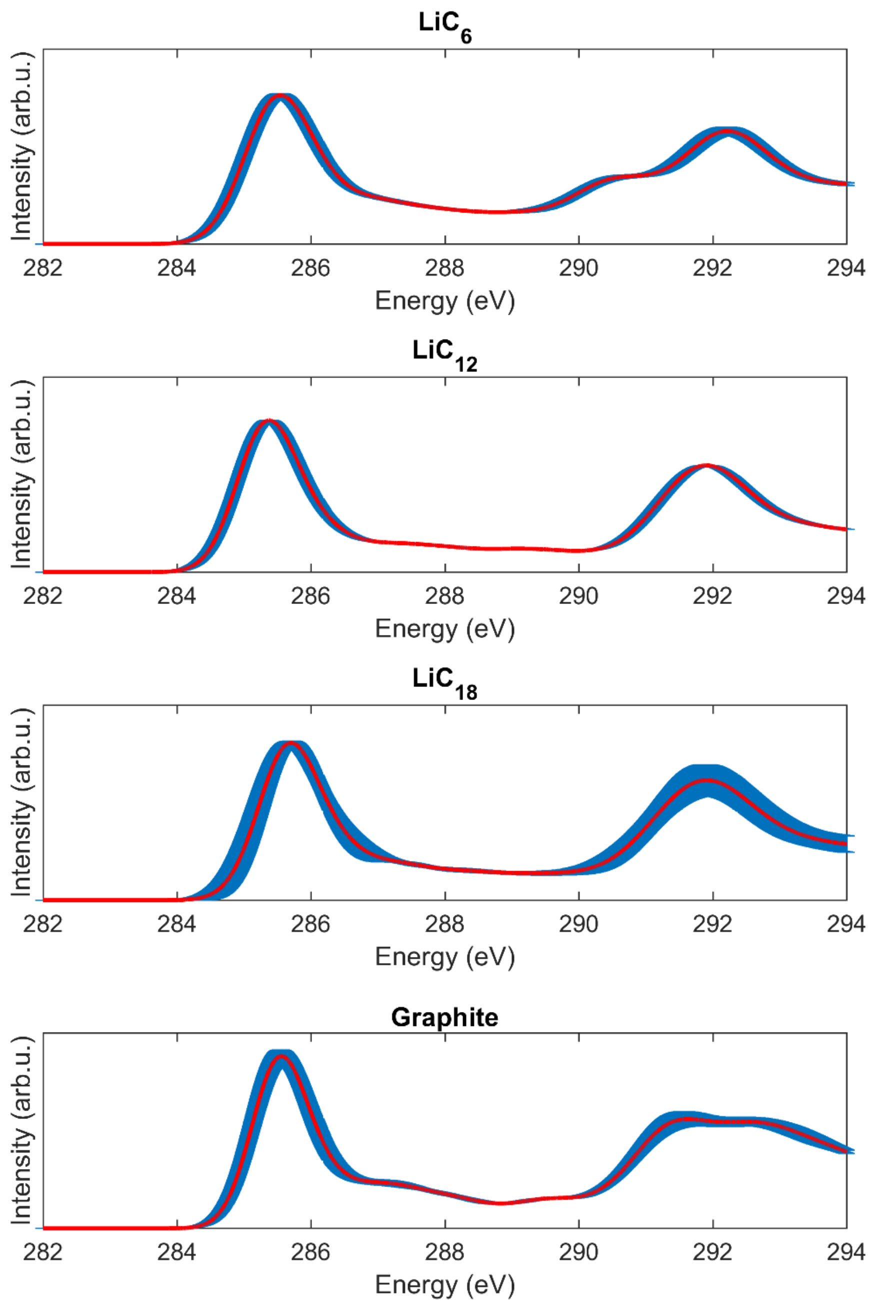

Supplementary Figure 11: Statistical averaged XAS Carbon K-edge spectra of graphite and Li-GICs. The shaded area represents the uncertainty from the standard deviation.
